# Drug-Coated Balloon Angioplasty and Debulking for the Treatment of Femoropopliteal In-Stent Restenosis: A Systematic Review and Meta-Analysis

**DOI:** 10.1155/2020/3076346

**Published:** 2020-06-10

**Authors:** Zhu Tong, Lianrui Guo, Lixing Qi, Shijun Cui, Xixiang Gao, Yang Li, Jianming Guo, Yongquan Gu

**Affiliations:** Department of Vascular Surgery, Xuanwu Hospital, Capital Medical University, Beijing 100053, China

## Abstract

The purpose of this article was to compare the efficiency and safety of drug-coated balloon angioplasty (DCB) and atherectomy with percutaneous transluminal angioplasty (PTA) in patients with femoropopliteal in-stent restenosis (ISR). Pubmed, Embase, and the Cochrane Central Register of Controlled Trials (CENTRAL) (all up to March 2019) were searched systematically. Trial sequential analysis (TSA) was conducted. 5 studies with 599 participants were included. Compared with PTA, DCB significantly increased the rate of patency (6 months: RR 1.65, 95% CI 1.30 to 2.09, *P* < 0.01; 12 months: RR 2.38, 95% CI 1.71 to 3.30, *P* < 0.01) and the rate freedom from target lesion revascularization (TLR) (6 months: RR 1.18, 95% CI 1.09 to 1.28, *P* < 0.01; 12 months: RR 1.56, 95% CI 1.33 to 1.82, *P* < 0.01) at 6 and 12 months follow-up, and the TSA results showed these outcomes were reliable. The rate of clinical improvement by ≥1 Rutherford category in the DCB group was higher than that in the PTA group (6 months: RR 1.35, 95% CI 1.03 to 1.75, *P* = 0.03; 12 months: RR 1.46, 95% CI 1.17 to 1.82, *P* < 0.01) at 6 and 12 months. There is no statistically difference of ABI, all-cause mortality, and incidence of amputation between DCB group and PTA group (MD 0.03, 95% CI -0.03 to 0.08, *P* = 0.40; RR 1.24, 95% CI 0.46 to 3.34, *P* = 0.67; RR 0.32, 95% CI 0.01 to 7.61, *P* = 0.48). Compared with PTA, the rate of patency and freedom from TLR in the laser atherectomy (LD) group was higher than that in the PTA group (patency: 6 months: RR 1.28, 95% CI 1.01 to 1.64, *P* < 0.05, 12 months: RR 2.25, 95% CI 1.14 to 4.44, *P* < 0.05; freedom from TLR: 6 months: RR 1.27, 95% CI 1.05 to 1.53, *P* = 0.01, 12 months: RR 1.59, 95% CI 1.12 to 2.25, *P* = 0.01) at 6 and 12 months follow-up. In conclusion, DCB and LD had superior clinical (freedom from TLR and clinical improvement) and angiographic outcomes (patency rate) compared with PTA for the treatment of femoropopliteal ISR. Moreover, DCB and LD had a low incidence of amputation and mortality and were relatively safe methods.

## 1. Introduction

Self-expanding nitinol stent is increasingly used for the treatment of symptomatic femoropopliteal arterial occlusive disease because of its reduction of stent fracture and other procedural complications of earlier devices. Despite these clear benefits, in-stent restenosis (ISR) remains a challenging clinical problem. Worldwide, more than 400000 stents are implanted in femoropopliteal arterial annually, 30%-40% of which will develop ISR within 2-3 years of implantation [1].

Different from de novo lesions closely associated with atherosclerosis, ISR lesions are predominantly caused by neointimal hyperplasia, which consists primarily of smooth muscle cells and hydrated collagen matrix [2]. And ISR lesions are usually long and highly calcified. Conventional endovascular therapies, such as percutaneous transluminal angioplasty (PTA) and repeat stenting, as methods of treating femoropopliteal ISR have no satisfactory clinical outcomes. Several prospective single-arm [3, 4] and retrospective trials [5, 6] have introduced methods of drug-coated balloon angioplasty (DCB, carrying antiproliferative drug, usually paclitaxel), or atherectomy (directional or laser) for the treatment of femoropopliteal ISR and had promising results. In recent years, some prospective controlled trials compared the effectiveness and safety of DCB, and atherectomy with PTA in the treatment of femoropopliteal ISR. However, the results are inconclusive [7–9].

In consideration of these inconclusive results, we carried out this meta-analysis of the prospective controlled trials including patients with femoropopliteal ISR treated with either DCB and atherectomy or PTA.

## 2. Materials and Methods

### 2.1. Protocol and Registration

Our meta-analysis was conducted according to the Preferred Reporting Items for Systematic reviews and meta-analyses (PRISMA) recommendations. A protocol for this meta-analysis has been registered on PROSPERO (http://www.crd.york.ac.uk/prospero) and the registration number is CRD42019128171.

### 2.2. Search Strategy

Pubmed, Embase, and the Cochrane Central Register of Controlled Trials (CENTRAL) (all up to March 2019) were searched according to the guidelines of the Cochrane Handbook, without language restrictions. The following subject headings and keywords: “restenosis” were used, “superficial femoral artery”, “popliteal artery”, “femoropopliteal artery” et al. A supplementary search of the reference lists from all retrieved trials and reviews was also performed. In case the articles were not available from databases, we directly contacted the corresponding authors by mail. All results were imported into Endnote X9 (Thomson Reuters, New York, USA) for the exclusion of duplicates, and subsequently, we screened the titles, abstracts, and full-texts of eligible trials.

### 2.3. Inclusion and Exclusion Criteria

Inclusion criteria were (1) prospective controlled studies; (2) patients with femoropopliteal ISR; (3) treatment methods of experiments were DCB or atherectomy; (4) a minimum follow-up of 6 months.

### 2.4. Endpoints and Data Extraction

The primary endpoints were patency at 6 and 12 months, and freedom from target lesion revascularization (TLR) at 6 and 12 months. The secondary endpoints included clinical improvement by ≥1 Rutherford category at 6 and 12months, ankle-brachial index (ABI) at 6 and 12 months, all-cause mortality at 6 and 12 months, and amputation. Data including date of publication, name of the first author, study type, patient characteristics (mean age, number of patients and sex ratio, basic diseases), inclusion criteria, exclusion criteria, and characteristics of ISR lesions. And the above endpoints were extracted from the eligible studies using a standard data extraction form.

Database search, eligibility evaluation, and data extraction were performed independently by two authors (database search was conducted by Zhu Tong and Lianrui Guo; eligibility evaluation was conducted by Xixiang Gao and Zhu Tong; data extraction was conducted by Lixing Qi, Shijun Cui); lack of consensus was resolved by a third author.

### 2.5. Data Synthesis and Statistical Analysis

Risk ratio (RR) with 95% confidence intervals (CIs) and standardized mean difference (SMD) with 95% CIs were used for the expression of dichotomous and continuous variables, respectively. Getdata software was used to get data on condition that data were presented as statistical graph only and were not procured from the corresponding author. A *P* value <0.05 was considered as statistically significant. The heterogeneity among trials was assessed with the Cochran's *Q*-statistic test and the *I*2 test. If *I*2 was more than 50% or *P* value (*Q*-test) was less than 0.05, we thought high heterogeneity existed and a random-effects model was adopted. Otherwise, a fixed effects model was used. Subgroup analysis was performed according to the treatment method (DCB or atherectomy) of experiment group to establish the derivation of heterogeneity. The statistical analysis was conducted using RevMan software (version 5.1; Cochrane Collaboration, Copenhagen, Denmark). Trial sequential analysis (TSA) was conducted to control the risks of type I and type II errors and calculate required information size (RIS) [10].

### 2.6. Risk of Bias

Two authors independently assessed the following seven categories of risk of bias according to the Cochrane guidelines, and lack of consensus was resolved in group discussions. The risk of bias was classified in the following seven categories: (1) random sequence generation, (2) allocation concealment, (3) blinding of participants and personnel, (4) blinding of outcome assessment, (5) incomplete outcome data, (6) selective outcome reporting, (7) other sources of bias. Each category can be graded into three levels: low risk, unclear risk, or high risk.

## 3. Results

### 3.1. Study Selection

**Figure 1:**
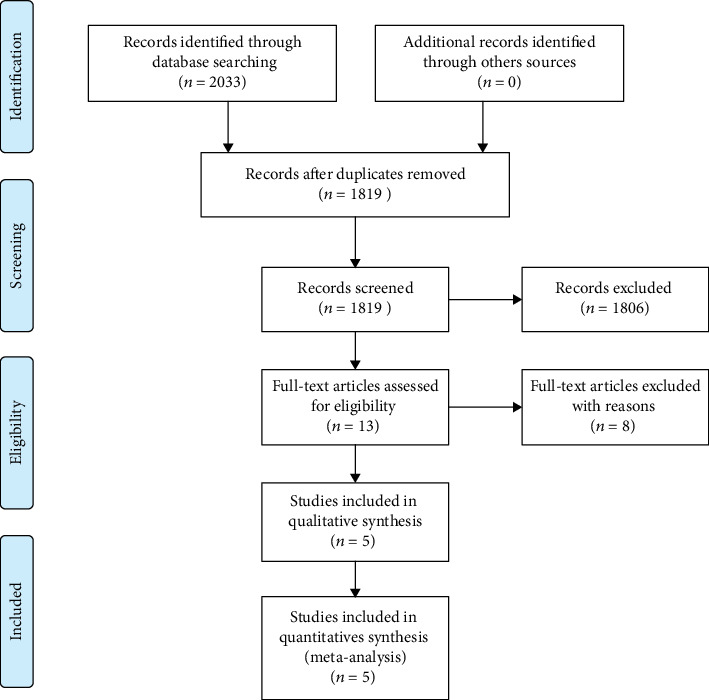
PRISMA flow chart.

2033 articles were identified initially. Finally, a total of 5 studies with 599 participants were included in this meta-analysis [7–9, 11, 12]. The detailed process of literature search is summarized in Figure 1.

### 3.2. Study Characteristics

4 studies compared the treatment method of DCB and PTA, 1 compared debulking and PTA. Patients included these studies were elderly, and most of them had disease of dyslipidemia and hypertension. The clinical and angiographic characteristics of patients between groups in each study were comparable. More details are summarized in Table 1.

### 3.3. Risk of Bias and Quality of Evidence

**Figure 2:**
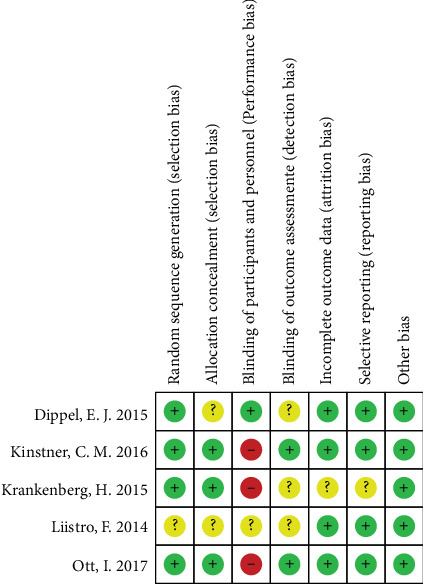
Risk of bias summary.

The risk of bias of included studies was showed in Figure 2.

### 3.4. Clinical Outcomes

#### 3.4.1. Patency

**Figure 3:**
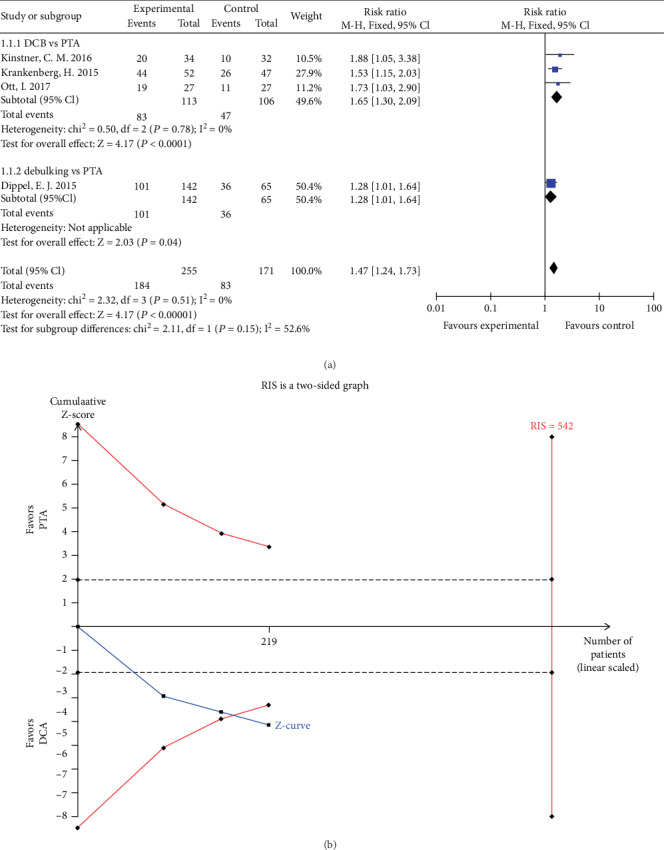
(a) Forest plot for patency at 6 months. M-H: Mantel–Haenszel test; Fixed: a fixed effects model; CI: confidence intervals. (b) TSA for patency at 6 months. TSA: trial sequential analysis.

Four studies reported vessel patency at 6 months follow-up. The rate of patency in the DCB group was significantly higher than that in the PTA group (RR 1.65, 95% CI 1.30 to 2.09, *P* < 0.01) using a fixed effects model (*I*2 = 0%, *P* = 0.78) (Figure 3(a)). TSA was conducted and the RIS was 542 participants. The cumulative Z curve (blue line) crossed the trial sequential monitoring boundaries (red inward slash) before the RIS has been reached (Figure 3(b)), indicating that positive result has been achieved in advance and further randomized controlled trials (RCTs) are unnecessary. The rate of patency in the debulking group was higher than that in the PTA group (RR 1.28, 95% CI 1.01 to 1.64, *P* < 0.05) (Figure 3(a)).

**Figure 4:**
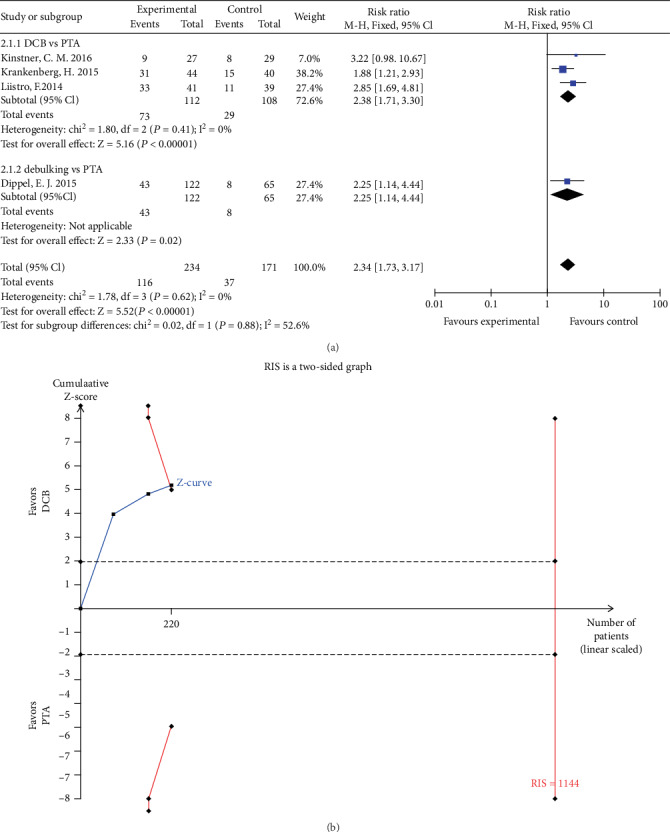
(a) Forest plot for patency at 12 months. M-H: Mantel–Haenszel test; Fixed: a fixed effects model; CI: confidence intervals. (b) TSA for patency at 6 months. TSA: trial sequential analysis.

Four studies reported vessel patency at 12 months follow-up. The rate of patency in the DCB group was significantly higher than that in the PTA group (RR 2.38, 95% CI 1.71 to 3.30, *P* < 0.01) using a fixed effects model (*I*2 = 0%, *P* = 0.41) (Figure 4(a)). TSA was conducted and the RIS was 1144 participants. The result of TSA indicating that positive result has been achieved in advance and further RCTs are unnecessary (Figure 4(b)). The rate of patency in the debulking group was higher than that in the PTA group (RR 2.25, 95% CI 1.14 to 4.44, *P* < 0.05) (Figure 4(a)).

### 3.5. Freedom from TLR

**Figure 5:**
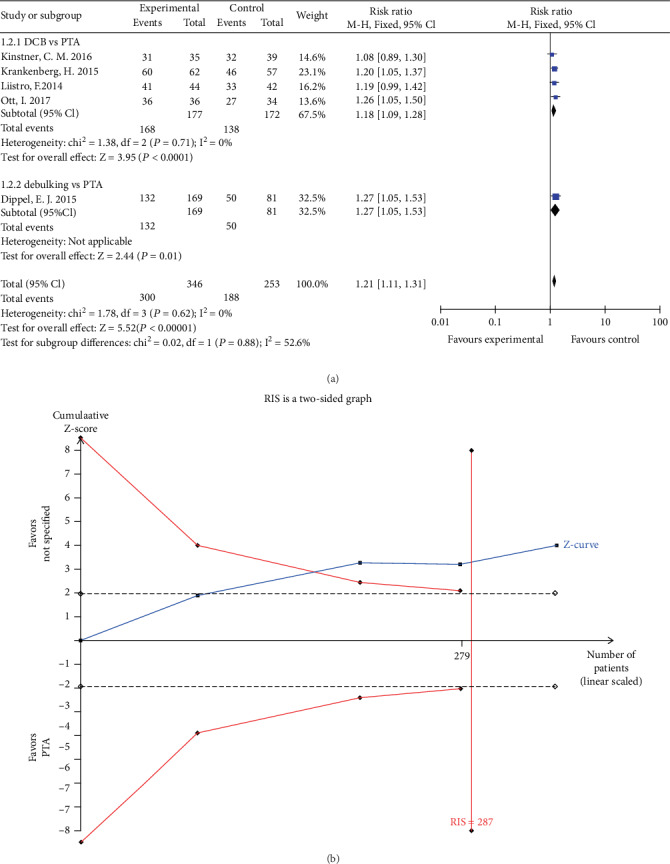
(a) Forest plot for freedom from TLR at 6 months. M-H: Mantel–Haenszel test; Fixed: a fixed effects model; CI: confidence intervals; TLR: target lesion revascularization. (b) TSA for freedom from TLR at 6 months. TSA: trial sequential analysis; TLR: target lesion revascularization.

Five studies reported freedom from TLR at 6 months follow-up. The rate of freedom from TLR in the DCB group was significantly higher than that in the PTA group (RR 1.18, 95% CI 1.09 to 1.28, *P* < 0.01) using a fixed effects model (*I*2 = 0%, *P* = 0.71) (Figure 5(a)). TSA was conducted and the RIS was 287 participants. The cumulative Z curve (blue line) crossed the trial sequential monitoring boundaries (red inward slash) and the RIS has been reached (Figure 5(b)). Consequently, we could think that our result is reliable and no more RCTs are needed. The rate of freedom from TLR in the debulking group was higher than that in the PTA group (RR 1.27, 95% CI 1.05 to 1.53, *P* = 0.01) (Figure 5(a)).

**Figure 6:**
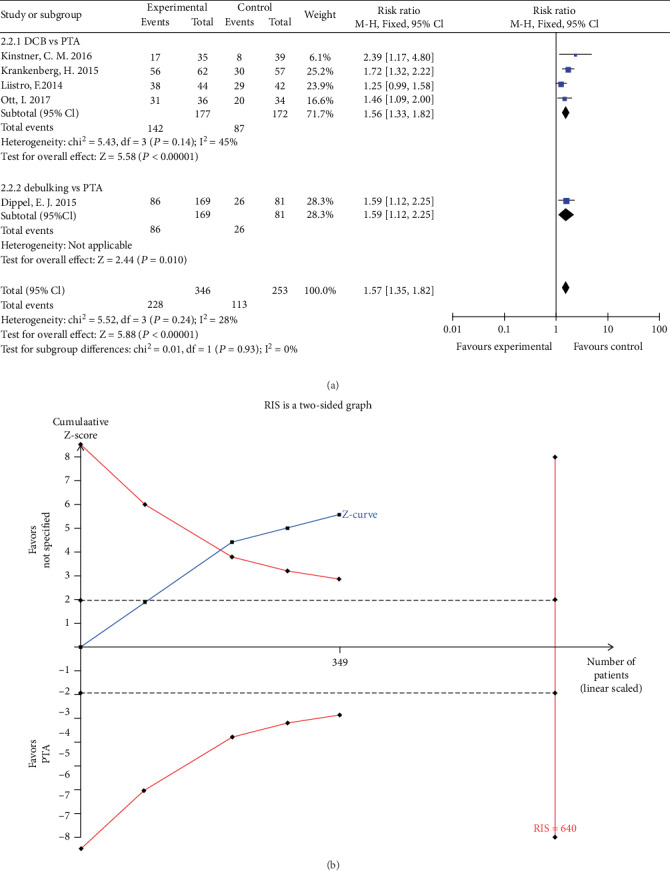
(a) Forest plot for freedom from TLR at 12 months. M-H: Mantel–Haenszel test, Fixed: a fixed effects model, CI: confidence intervals, TLR: target lesion revascularization. (b) TSA for freedom from TLR at 12 month. TSA: trial sequential analysis, TLR: target lesion revascularization.

Five studies reported freedom from TLR at 12 months follow-up. The rate of freedom from TLR in the DCB group was significantly higher than that in the PTA group (RR 1.56, 95% CI 1.33 to 1.82, *P* < 0.01) using a fixed effects model (*I*2 = 45%, *P* = 0.14) (Figure 6(a)). TSA was conducted and the RIS was 640 participants. The result of TSA indicating that positive result has been achieved in advance, and further RCTs are unnecessary (Figure 6(b)). The rate of freedom from TLR in the debulking group was higher than that in the PTA group (RR 1.59, 95% CI 1.12 to 2.25, *P* = 0.01) (Figure 6(a)).

### 3.6. ABI

**Figure 7:**
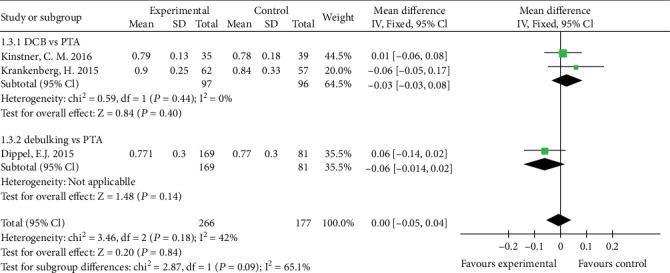
Forest plot for ABI at 6 month. IV: inverse variance; Fixed: a fixed effects model; CI: confidence intervals; ABI: ankle-brachial index.

**Figure 8:**
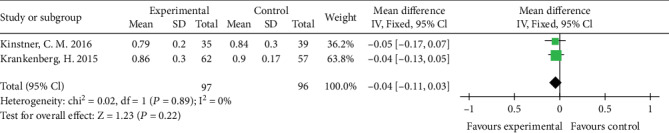
Forest plot for ABI at 12 month. IV: inverse variance; Fixed: a fixed effects model; CI: confidence intervals, ABI: ankle-brachial index.

Three studies reported ABI at 6 months and two studies reported ABI at 12 months. There is no statistical difference of ABI between DCB group and PTA group (MD 0.03, 95% CI -0.03 to 0.08, *P* = 0.40) using a fixed effects model (*I*2 = 0%, *P* = 0.44) at 6 months (Figure 7) and (MD -0.04, 95% CI -0.11 to 0.03, *P* = 0.22) using a fixed effects model (*I*2 = 0%, *P* = 0.89) at 12 months (Figure 8).

There is no statistical difference of ABI between the debulking group and PTA group (MD -0.06, 95% CI -0.14 to 0.02, *P* = 0.14) at 6 months (Figure 7).

### 3.7. Clinical Improvement by ≥1 Rutherford Category

**Figure 9:**
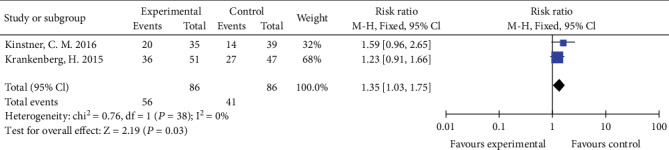
Forest plot for clinical improvement at 6 months. M-H: Mantel–Haenszel test; Fixed: a fixed effects model, CI: confidence intervals.

**Figure 10:**
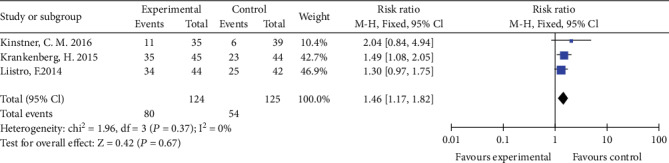
Forest plot for clinical improvement at 12 months. M-H: Mantel–Haenszel test; Fixed: a fixed effects model; CI: confidence intervals.

Two studies reported clinical improvement by ≥1 Rutherford category at 6 months, and three studies reported clinical improvement by ≥1 Rutherford category at 12 months. The rate of clinical improvement by ≥1 Rutherford category in the DCB group was significantly higher than that in the PTA group (RR 1.35, 95% CI 1.03 to 1.75, *P* = 0.03 and RR 1.46, 95% CI 1.17 to 1.82, *P* < 0.01) using a fixed effects model (*I*2 = 0%, *P* = 0.38 and *I*2 = 0%, *P* = 0.56) at 6 and 12 months (Figures 9 and 10).

## 4. Adverse Events

### 4.1. All-Cause Mortality

**Figure 11:**
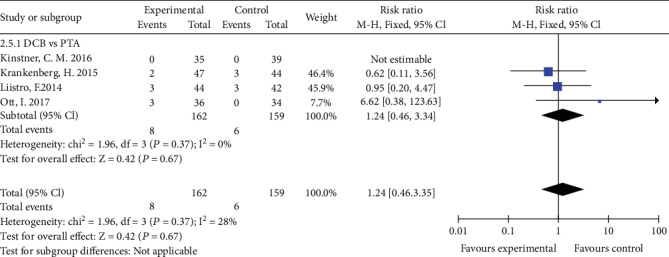
Forest plot for all-cause mortality. M-H: Mantel–Haenszel test; Fixed: a fixed effects model; CI: confidence intervals.

Four studies reported all-cause mortality. There was no statistical difference of all-cause mortality between DCB group and PTA group (RR 1.24, 95% CI 0.46 to 3.34, *P* = 0.67) using a fixed effects model (*I*2 = 0%, *P* = 0.37) (Figure 11).

### 4.2. Amputation

**Figure 12:**
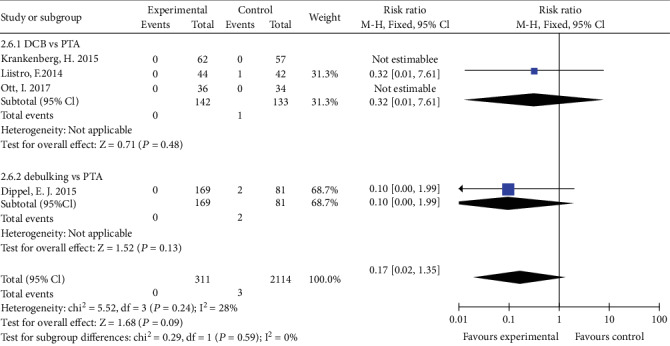
Forest plot for amputation. M-H: Mantel–Haenszel test; Fixed: a fixed effects model; CI: confidence intervals.

Four studies reported the incidence of amputation. There was no statistically difference of incidence of amputation between DCB group and PTA group (RR 0.32, 95% CI 0.01 to 7.61, *P* = 0.48) and between debulking group and PTA group (RR 0.17, 95% CI 0.02 to 1.35, *P* = 0.09) using a fixed effects model (Figure 12).

### 4.3. Publication Bias

Egger's funnel plot showed no publication bias (Additional file).

## 5. Discussion

In the present meta-analysis, we evaluated the efficacy and safety of two new therapies (DCB and debulking) versus PTA in patients suffering from femoropopliteal ISR. DCB, firstly used in coronary arteries, delivers antiproliferative drug, usually paclitaxel at the site of lesions, which could inhibit smooth muscle cell replication and proliferation and therefore reduce the incidence of restenosis [13]. In the following years, researchers found the superiority of DCB for the treatment of coronary ISR to PTA [14, 15]. Because of the ability of locally drug delivery and promising results in the treatment of coronary ISR, DCB had attracted attention and was tentatively used in the treatment of femoropopliteal ISR.

Four studies comparing DCB with PTA were included in this meta-analysis. The key findings are that in patients with femoropopliteal ISR. (1) In comparison to PTA, DCB had a higher patency rate at 6 and 12 months follow-up, and TSA results showed that our outcomes were reliable and no more RCTs are required. (2) DCB increased the rate of freedom from TLR significantly compared with PTA at 6 and 12 months follow-up, and TSA results showed that our outcomes were reliable and no more RCTs are required. (3) Clinical improvement in the DCB group was superior to that in the PTA group. (4) The ABI in two groups was both below normal (but above baseline), and no difference was found between two groups at 6 and 12 months follow-up, indicating that DCB did not significantly increase the ABI and improve Ischemic condition, which seemed contradictory to clinical outcomes (freedom from TLR and clinical improvement) and angiographic outcomes (patency rate). (5) There was no difference in mortality, incidence of amputation, and incidence of all-cause adverse events between two groups.

Debulking therapy includes directional atherectomy (DA) and laser atherectomy (LD). Directional atherectomy works through cutting blades which resects the obstructive plaque longitudinally [16]. Different from directional atherectomy, laser atherectomy works through laser-guided photoablation which breaks down and vaporizes the obstructive plaque [17]. Debulking therapy has unique advantages used in the treatment of ISR such as significant removing of hyperplastic neointima, which is the predominant composition of ISR lesions, enlarging the lumen volume and recanalizing the vessel. To now, EXCITE-ISR is the unique prospective controlled study comparing the efficacy of debulking and PTA. The result showed that at 6 months compared with PTA, the method of LD had higher rate of patency (71.1% vs 56.4%, *P* = 0.004) and lower rate of TLR (20.2% vs 36.3%, *P* = 0.003). However, the same as DCB, LD did also not significantly increase the ABI and improve Ischemic condition.

Our findings are in accord with the results of the study of Shahab Hajibandeh et al. which has a conclusion that compared with PTA, DCB and LD both confer improved outcomes [18]. Although DCB has a distinct superiority, Charles Nicolais et al. thought it is not the first choice for the treatment of ISR. Their findings suggested that repeat stenting with second-generation drug-eluting stents (DES) is more likely to have optimal outcomes. DCB might only be an effective alternative on condition that repeat stenting is not preferable [19]. However, to now, there are no trials to compare the efficiency and safety of DCB and repeat stenting with DES. So more high-quality RCTs are needed to evaluate these two methods.

According to the findings of this meta-analysis, we have some questions.

First, although DCB and LD could significantly increase the rate of patency and freedom from TLR, their shortcomings are also not negligible. Firstly, DCB is still a method of balloon angioplasty and for the occupation of substantial hyperplastic tissues, its antiproliferative effect cannot be effectively exerted. Next, DCB alone has certain of procedural failure that leads to the application of bail stenting especially in complex diseases [20]. Again, simple debulking therapy injures vascular endothelium and leads to smooth muscle cell proliferation, which caused a high recurrence rate. There is a complementarity between DCB and debulking therapy. The combination of DA or LD with subsequent DCB may have the advantage of removal occupation and antiproliferative effect. In 2013, Gandini et al. conducted a randomized controlled trial (RCT) comparing the efficacy of LD+DCB with DCB, which is the only RCT to date [21]. The results indicated that LD+DCB significantly increased the rate of patency (6 months: 91.7% vs 58.3%, *P* = 0.01; 12 months: 66.7% vs 37.5%, *P* = 0.01) and decreased the rate of TLR (12 months: 16.7% vs 50%, *P* = 0.01) compared with DCB alone. In 2013, Sixt et al. retrospectively evaluated the efficacy of DA+DCB with DA [22]. The results indicated that DA+DCB significantly increased the rate of patency at 12 months (84.7% vs 43.8%, *P* < 0.01) compared with DA alone. In 2017, Kokkinidis, D. G. et al. retrospectively evaluated the efficacy of LD+DCB with LA [23]. The results indicated that LD+DCB significantly increased the rate of patency (86.7% vs 56.9%, *P* < 0.01) and decreased the rate of TLR (72.5% vs 50.5%, *P* < 0.05) at 12 months compared with LD alone. These results all showed the possible superior of the combination of debulking with DCB. So a number of large-scale RCTs are needed to further evaluate this combined method.

Second, our meta-analysis showed the inconsistence of ABI outcome with other clinical and angiographic outcomes. This inconsistency may be caused by the lack of adequate trials and sample size and the fact that only two studies with 193 patients reported ABI data.

Third, the pooled results showed that DCB, LD, and PTA had a very low incidence of amputation and mortality, and no statistical difference was found between them, indicating DCB and LD are relatively safe methods. However, a study found LD+DCB lowered the incidence of major amputation (8% vs 46%, *P* < 0.01) and mortality (12% vs 37%, *P* < 0.05) compared with DCB alone in diabetic foot patients with critical limb ischemia. We found that the ISR in the study of Gandini et al. were all totally occluded, and the diseases were more complex and serious. So I think more trials are needed to evaluate the safety of LD+DCB, DCB alone, and debulking alone for patients with complex diseases and totally occluded ISR.

The present analysis has several limitations. First, there were small number of RCTs (5 trials with 599 participants) included in the meta-analysis. Second, Tosaka et al. classified FP-ISR into three types: class I: focal ISR (≤50 mm length), class II: diffuse ISR (≥50 mm in length), class III: totally occluded ISR [24]. The ISR Tosaka classification of patients in individual trials consisted of class I, class II, and class III. However, the trials did not conduct subgroup analysis according to the ISR Tosaka classification. Third, it is unclear whether the bailout stent was counted as TLR in some trials (PACUBA, DEBATE-ISR, and ISAR-PEBIS).

## 6. Conclusion

In conclusion, our meta-analysis showed that DCB and LD had superior clinical (freedom from TLR and clinical improvement) and angiographic outcomes (patency rate) compared with PTA for the treatment of femoropopliteal ISR. Moreover, DCB and LD had a low incidence of amputation and mortality and were relatively safe methods. However, considering their inherent shortcomings, the combination of DA or LD with DCB was attempted to be used and had an encouraging result. Thus, a number of large-scale RCTs are needed to further evaluate the efficiency and safety of this combined method.

## Figures and Tables

**(a):** 

		Hypertension	DM	CAD	Dyslipidemia	Age, *y*	*n*	Male gender	Lesion length, mm	Rutherford class	ISR Tosaka classification	ABI
Ott, I. 2017 ISAR-PEBIS	DCB	33 (92%)	12 (33%)	17 (47%)	35 (97%)	70 ± 10	36	67%	132 ± 65	Class 2: 1; class 3: 34; class 4: 0; class 5: 1	Class 1: 10; class 2: 13; class 3: 13	0.6 ± 0.3
	PTA	30 (88%)	12 (35%)	16 (47%)	33 (97%)	68 ± 10	34	70%	146 ± 69	Class 2: 0; class 3: 33; class 4: 0; class 5: 1	Class 1: 17; class 2: 7; class 3: 10	0.7 ± 0.2
Kinstner, C. M. 2016 PACUBA	DCB	26 (79%)	17 (52%)	12 (36%)		68.1 ± 9.2	35	57%	173 ± 113	Class 2: 3; class 3: 32	Class 1: 8; class 2: 16; class 3: 11	0.65 ± 0.16
	PTA	27 (79%)	13 (38%)	14 (41%)		68.3 ± 0.4	39	59%	184 ± 88	Class 2: 8; class 3: 30	Class 1: 2; class 2: 26; class 3: 11	0.65 ± 0.16
Krankenberg, H. 2015 FAIR	DCB	52 (83.9%)	28 (45.2%)	26 (41.9%)		69 ± 8	62	53.2%	82.3 ± 70.9	Class 2: 27; class 3: 32; class 4: 1; class 5: 2	Class 1: 16; class 2: 32; class 3: 14	0.63 ± 0.27
	PTA	53 (93.0%)	17 (29.8)	22 (38.6%)		67 ± 9	57	70.2%	81.1 ± 66.2	Class 2: 27; class 3: 24; class 4: 6; class 5: 0	Class 1: 16; class 2: 30; class 3: 11	0.64 ± 0.25
Dippel, E. J. 2015 EXCITE	LD+PTA	95.8	47.0	64.3		68.5 ± 9.8	169	62.7%	196 ± 120			
	PTA	95.8	47.0	68.8		67.8 ± 10.3	81	61.7%	193 ± 119			
Liistro, F. 2014 DEBATE-	DCB	39 (88.6%)	44 (100%)	9 (20.5%)		74 ± 11	44	72.7%		Class ≥4: 33	Class 1: 7; class 2: 15; class 3: 22	0.32 ± 0.11
	PTA	38 (90.5%)	42 (100%)	12 (28.6%)		76 ± 7	42	54.8%		Class ≥4: 28	Class 1: 6; class 2: 8; class 3: 28	0.36 ± 0.9

**(b):** 

	Design	Year	Inclusion criteria	Exclusion criteria	Outcome	Follow-up month
Ott, I. 2017	Dual-center, prospective, randomized, active-controlled	2010.4-2013.12	SFA ISR symptomatic ISR >70% or occlusion of SFA at the stented site	Acute ischemia and/or acute thrombosis of the SFA, untreated ipsilateral iliac or popliteal artery stenosis >70%, severe renal insufficiency, life expectancy <1 year, and any contraindication to study medications	Percentage diameter stenosis (DS); binary restenosis rate; TLR; target vessel thrombosis, ipsilateral amputation, or bypass surgery of the affected limb; and all-cause mortality at 24 months	6 and 24
Kinstner, C. M. 2016	Dual- center, prospective, single-blind, randomized	2010.11-2012.12	SFA and popliteal artery ISR; age>50 years; symptomatic ISR >50%	Inability to give written informed consent; known allergy, hypersensitivity, or intolerance to radiologic contrast media, aspirin, clopidogrel or ticlopidine, and paclitaxel; and creatinine >2.5 mg/dl	Primary patency, technical success, TLR; ABI; sustained clinical improvement by ≥1 Rutherford category	6 and 12
Krankenberg, H. 2015	Multicenter, prospective, block-randomized, nonblinded	2010.1-2012.11	SFA ISR of up to 20 cm in length.	Untreated ipsilateral iliac artery stenosis, ongoing dialysis treatment, and treatment with oral anticoagulants other than antiplatelet agents	Binary restenosis rate; primary angiographic success; freedom from TLR; ABI; sustained clinical improvement by ≥1 Rutherford category; major adverse vascular events	6 and 12
Dippel, E. J. 2015	Multicenter, prospective, randomized, controlled	2011.6-2012.2	Femoropopliteal ISR, symptomatic ISR >50%,		Freedom from TLR; TLR; ABI; Rutherford score; all-cause death; amputation	6 and 12
Liistro, F. 2014	Single-site prospective, controlled	2010.1-2011.12	Diabetic patients with ISR of the superficial femoral and proximal popliteal arteries		Binary restenosis rate; TLR, major adverse events	6 and 12

DCB: drug-coated balloon angioplasty; PTA: percutaneous transluminal angioplasty; LD: laser debulking; DM: diabetes mellitus; CAD: coronary artery disease; ISR: in-stent restenosis; ABI: indicates ankle brachial index; SFA: superficial femoral artery.

## Supplementary Materials

Supplementary Materials Figure S1: (funnel plot of patency at 6 months), Figure S2: (funnel plot of patency at 12 months), Figure S3: (funnel plot of freedom from TLR at 6 months), Figure S4: (funnel plot of freedom from TLR at 12 months), Figure S5: (funnel plot of clinical improvement at 6 months), Figure S6: (funnel plot of clinical improvement at 12 months), Figure S7: (funnel plot of ABI at 6 months), Figure S8: (funnel plot of ABI at 12 months), Figure S9: (funnel plot of all-cause mortality), Figure S10: (funnel plot of amputation), PRISMA flow diagram, and Table S1: (8 full-text excluded articles).
