# Supplementary material for: Drug-Coated Balloon Angioplasty and Debulking for the Treatment of Femoropopliteal In-Stent Restenosis: A Systematic Review and Meta-Analysis
Source: Biomed Res Int. 2020 Jun 10;2020:3076346. doi: 10.1155/2020/3076346 (PMC7303732; doi:10.1155/2020/3076346)
Supplement: Supplementary Materials — Figure S1: (funnel plot of patency at 6 months), Figure S2: (funnel plot of patency at 12 months), Figure S3: (funnel plot of freedom from TLR at 6 months), Figure S4: (funnel plot of freedom from TLR at 12 months), Figure S5: (funnel plot of clinical improvement at 6 months), Figure S6: (funnel plot of clinical improvement at 12 months), Figure S7: (funnel plot of ABI at 6 months), Figure S8: (funnel plot of ABI at 12 months), Figure S9: (funnel plot of all-cause mortality), Figure S10: (funnel plot of amputation), PRISMA flow diagram, and Table S1: (8 full-text excluded articles). [file 3076346.f1.docx]

**Supplementary Material**

**Drug-coated balloon angioplasty and debulking for the treatment of femoropopliteal in-stent restenosis**


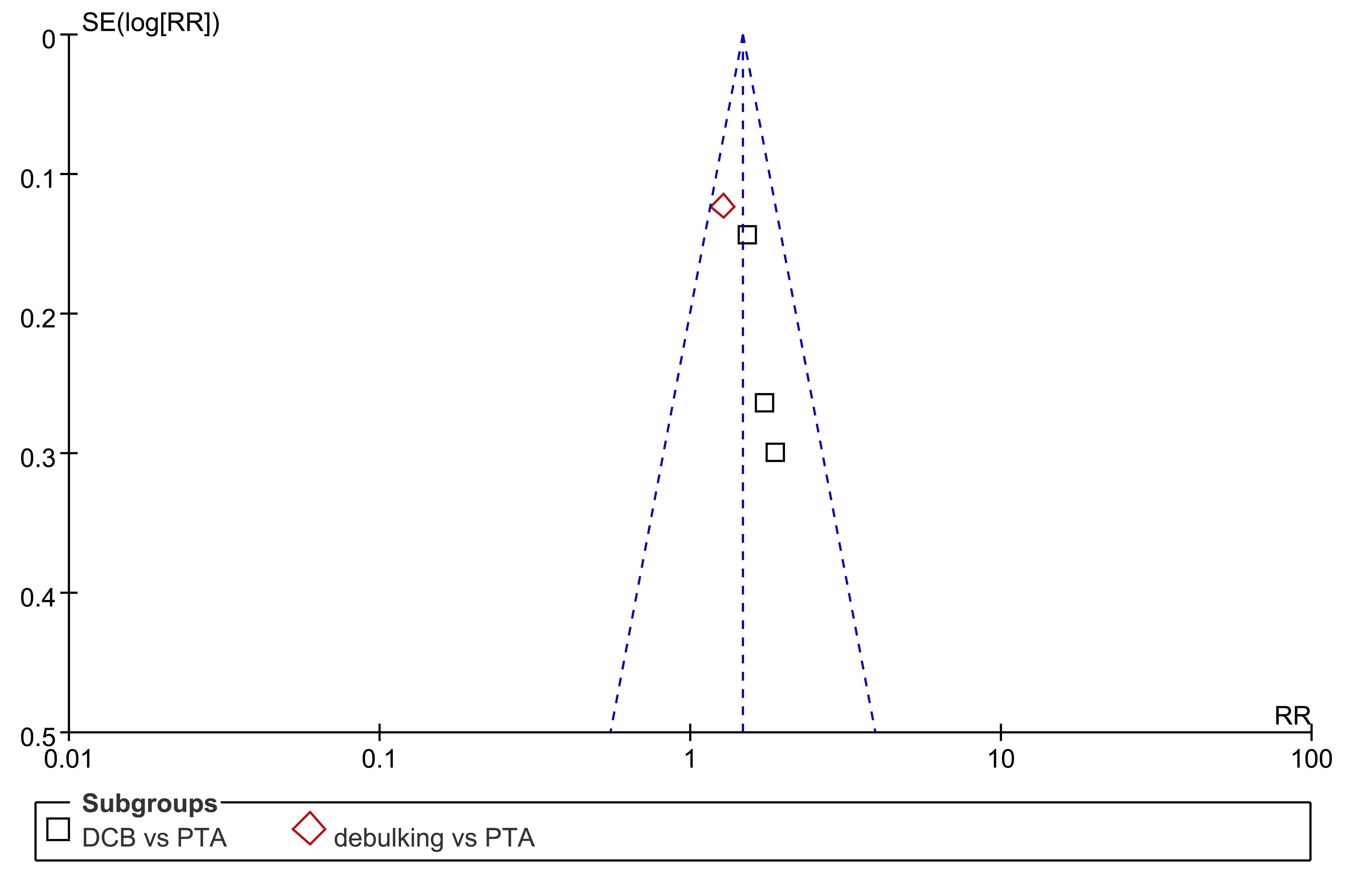


Figure S1 funnel plot of patency at 6 month


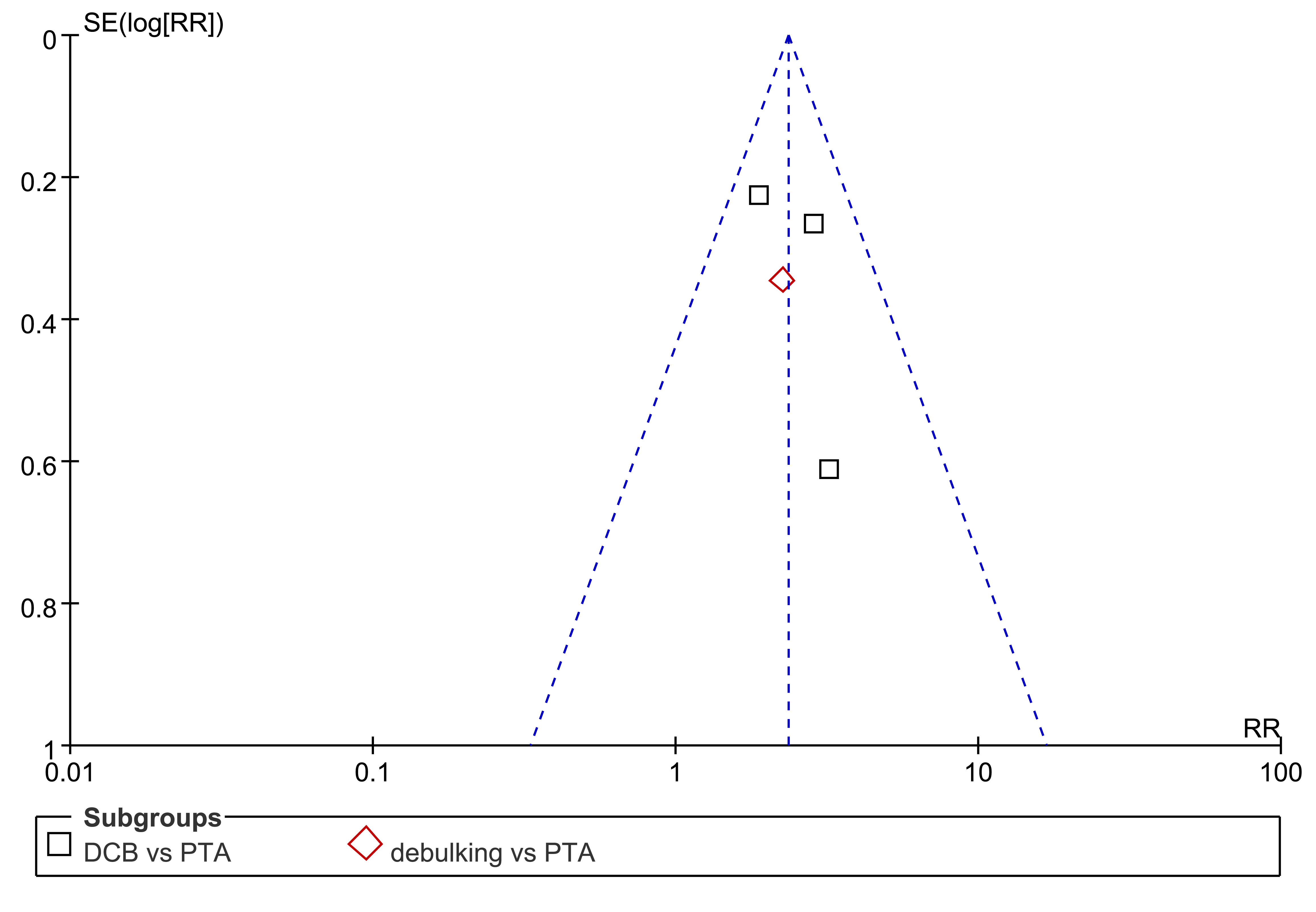


Figure S2 funnel plot of patency at 12 month


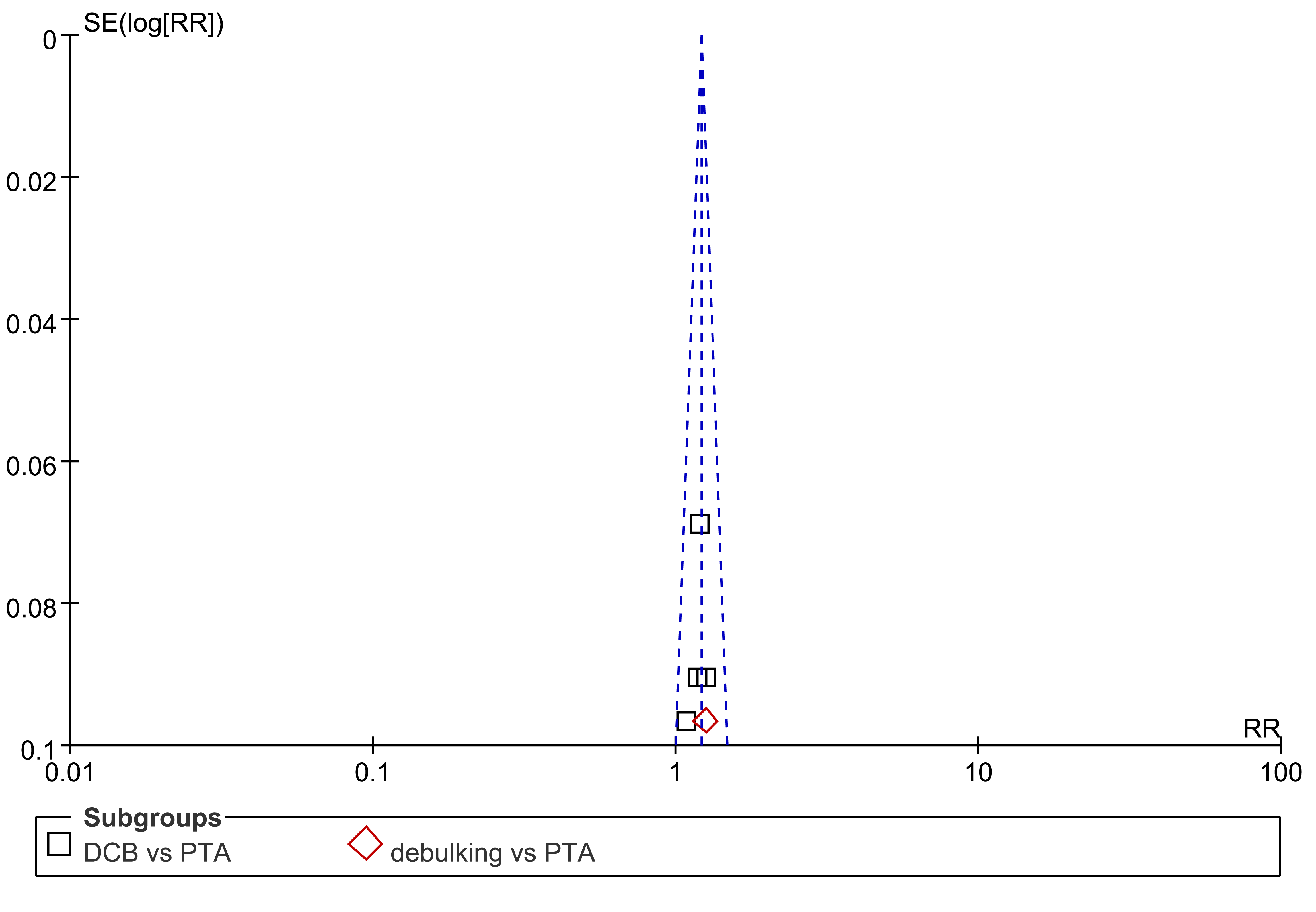


Figure S3 funnel plot of freedom from TLR at 6 month


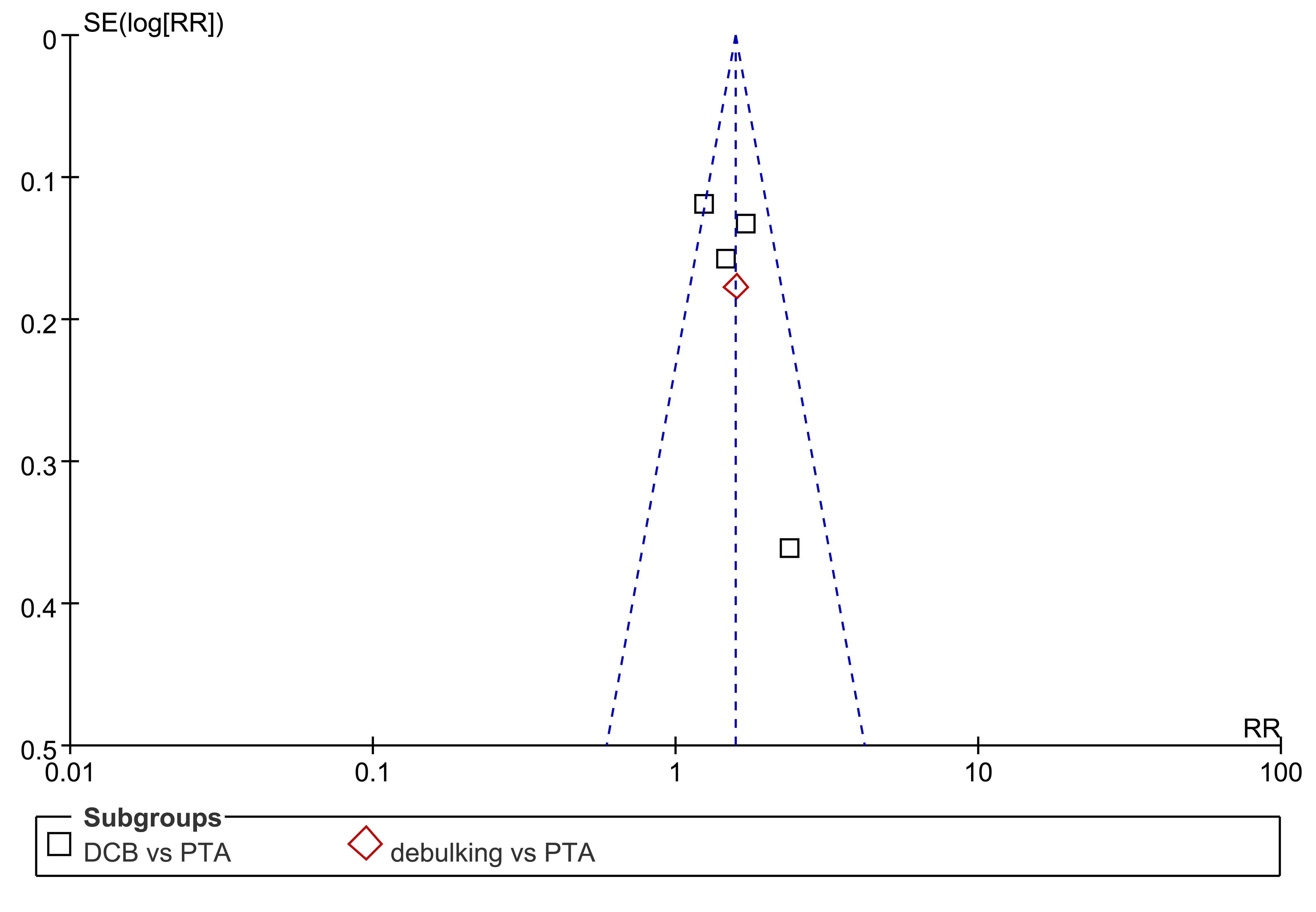


Figure S4 funnel plot of freedom from TLR at 12 month


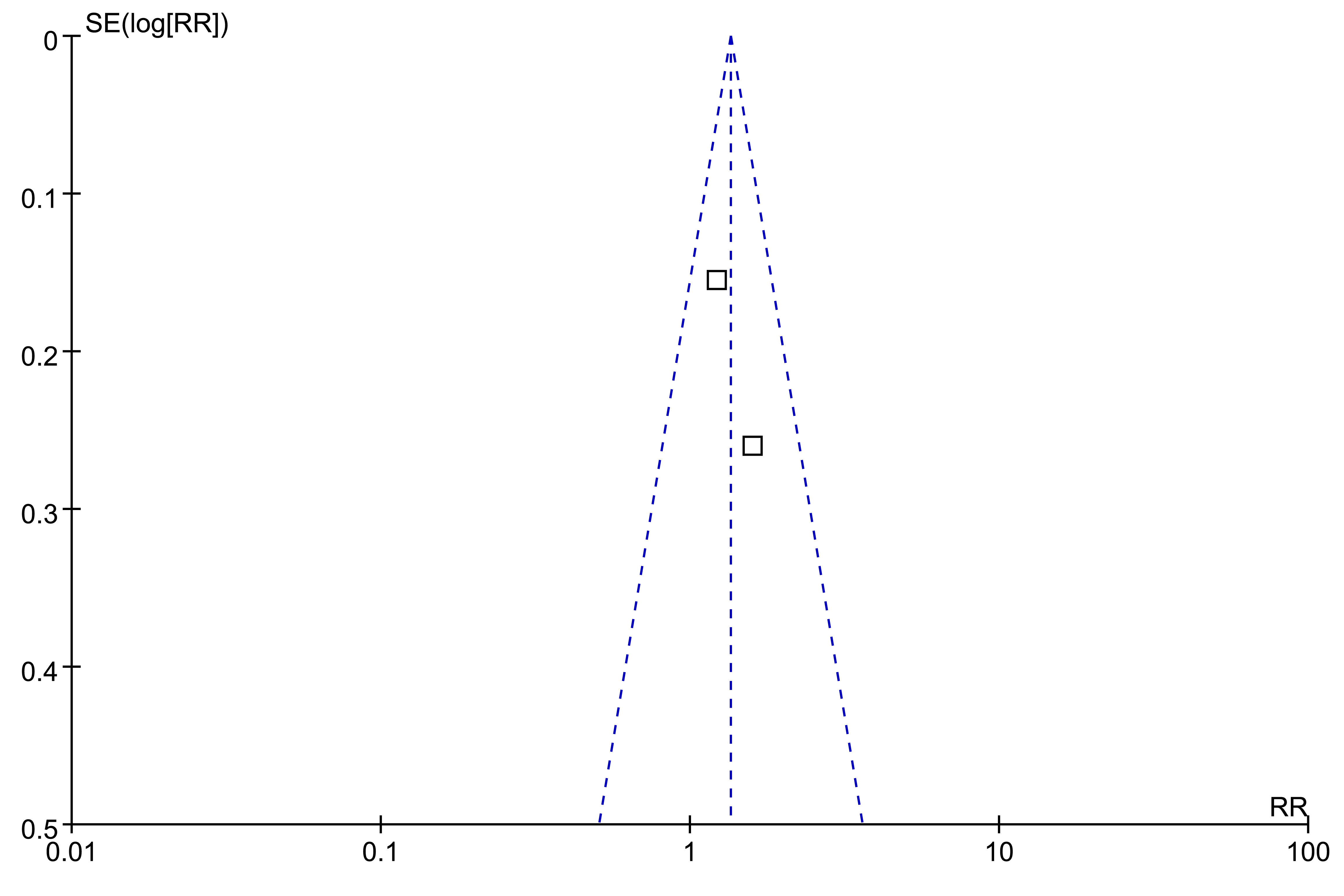


Figure S5 funnel plot of clinical improvement at 6 month


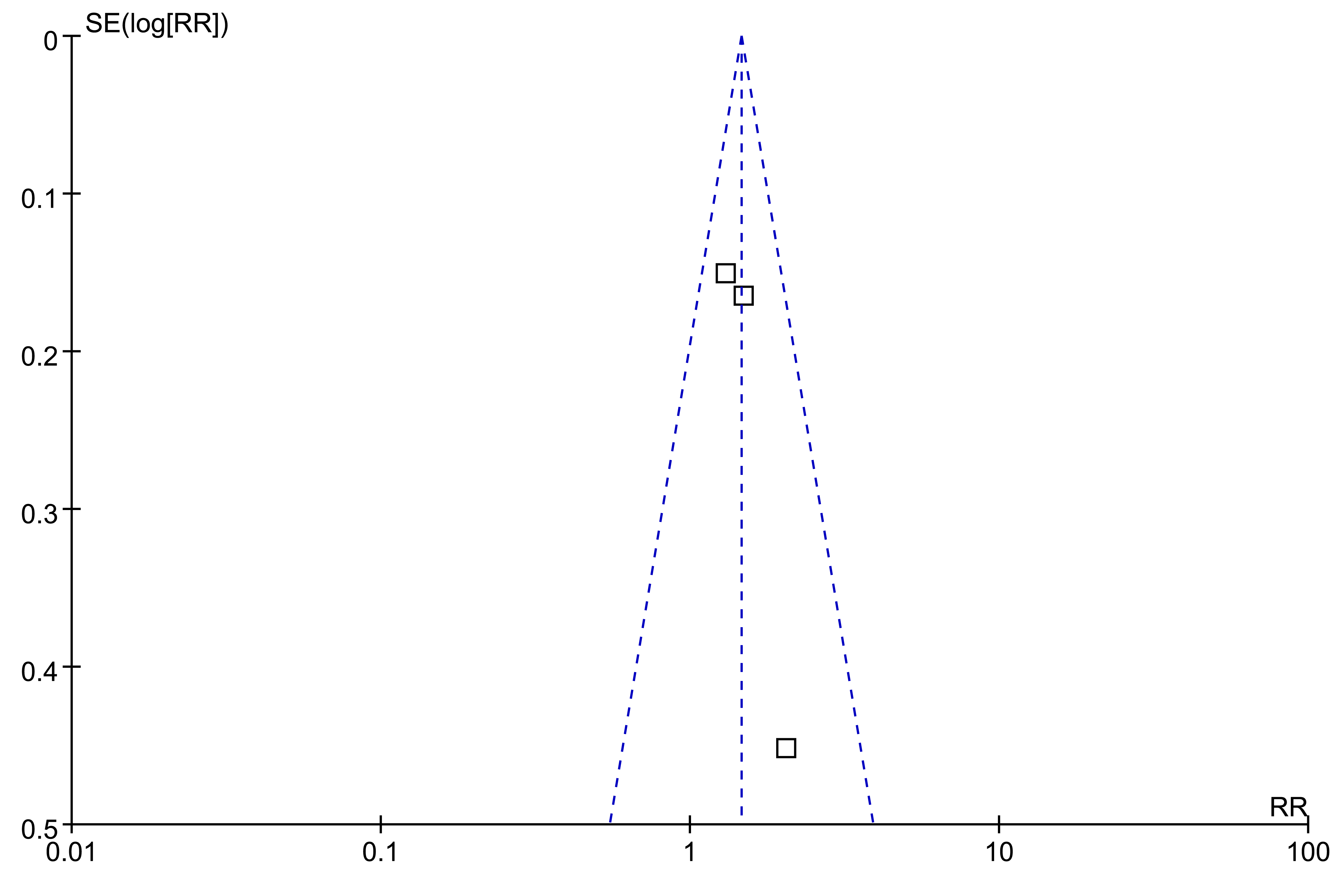


Figure S6 funnel plot of clinical improvement at 12 month


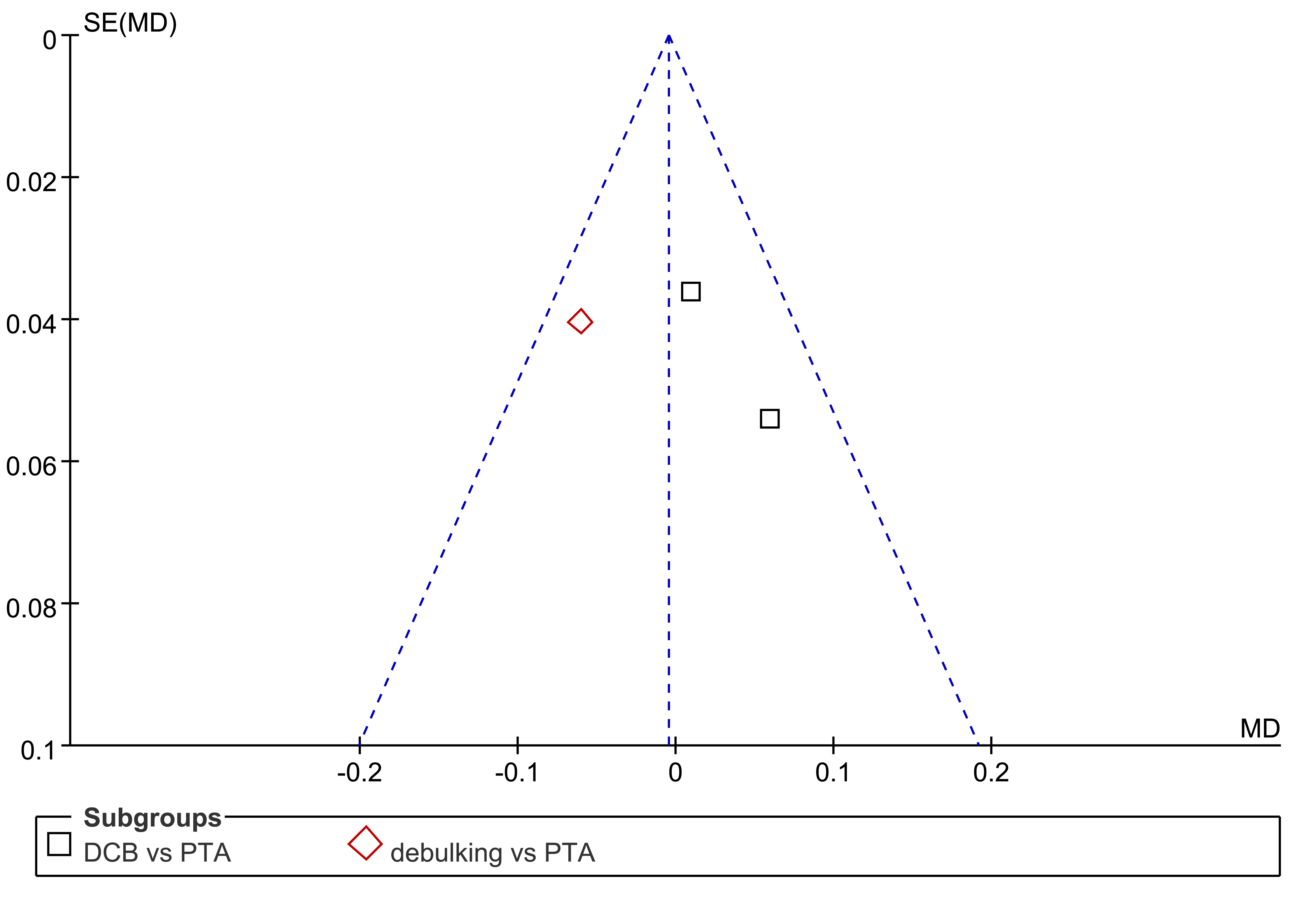


Figure S7 funnel plot of ABI at 6 month


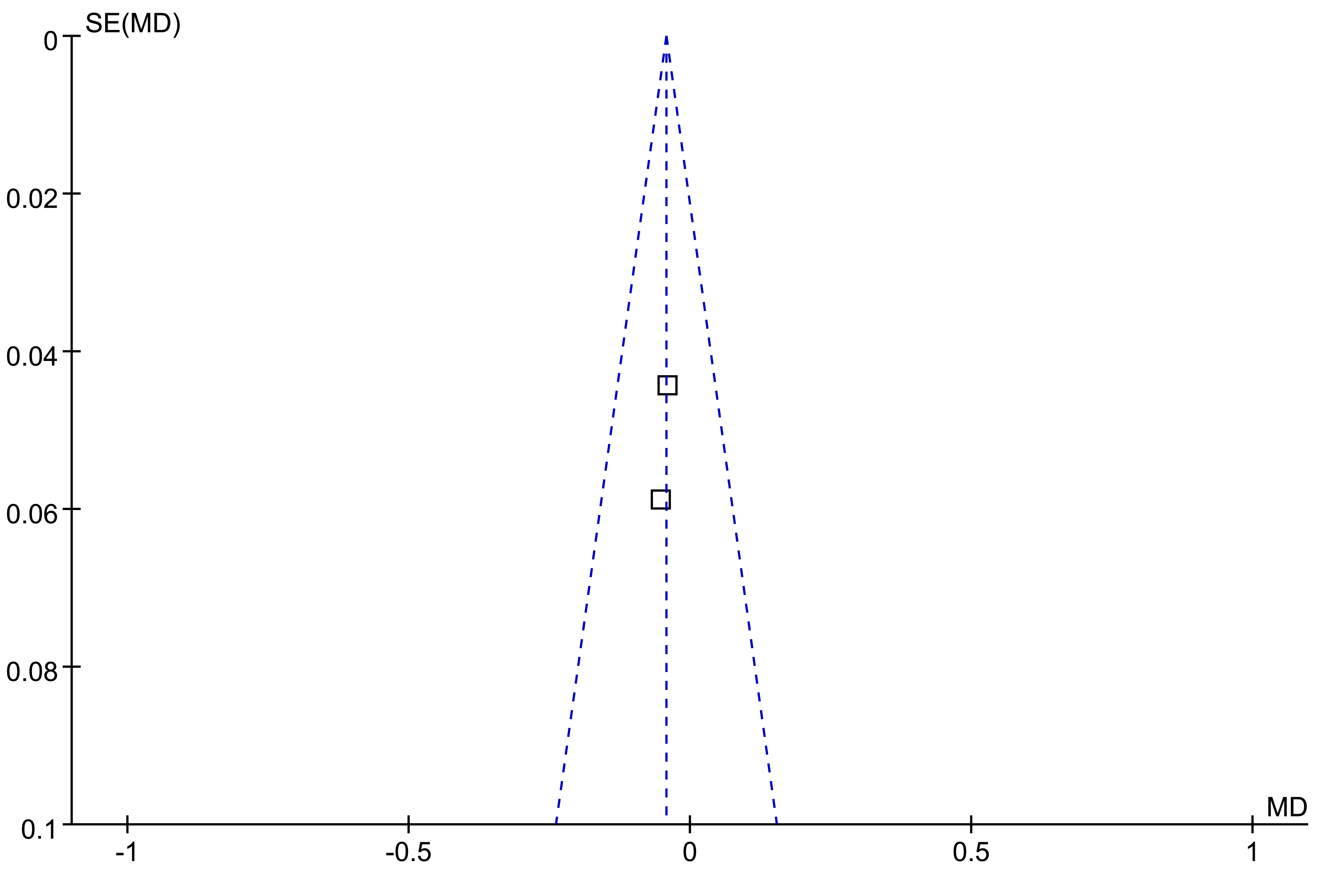


Figure S8 funnel plot of ABI at 12 month


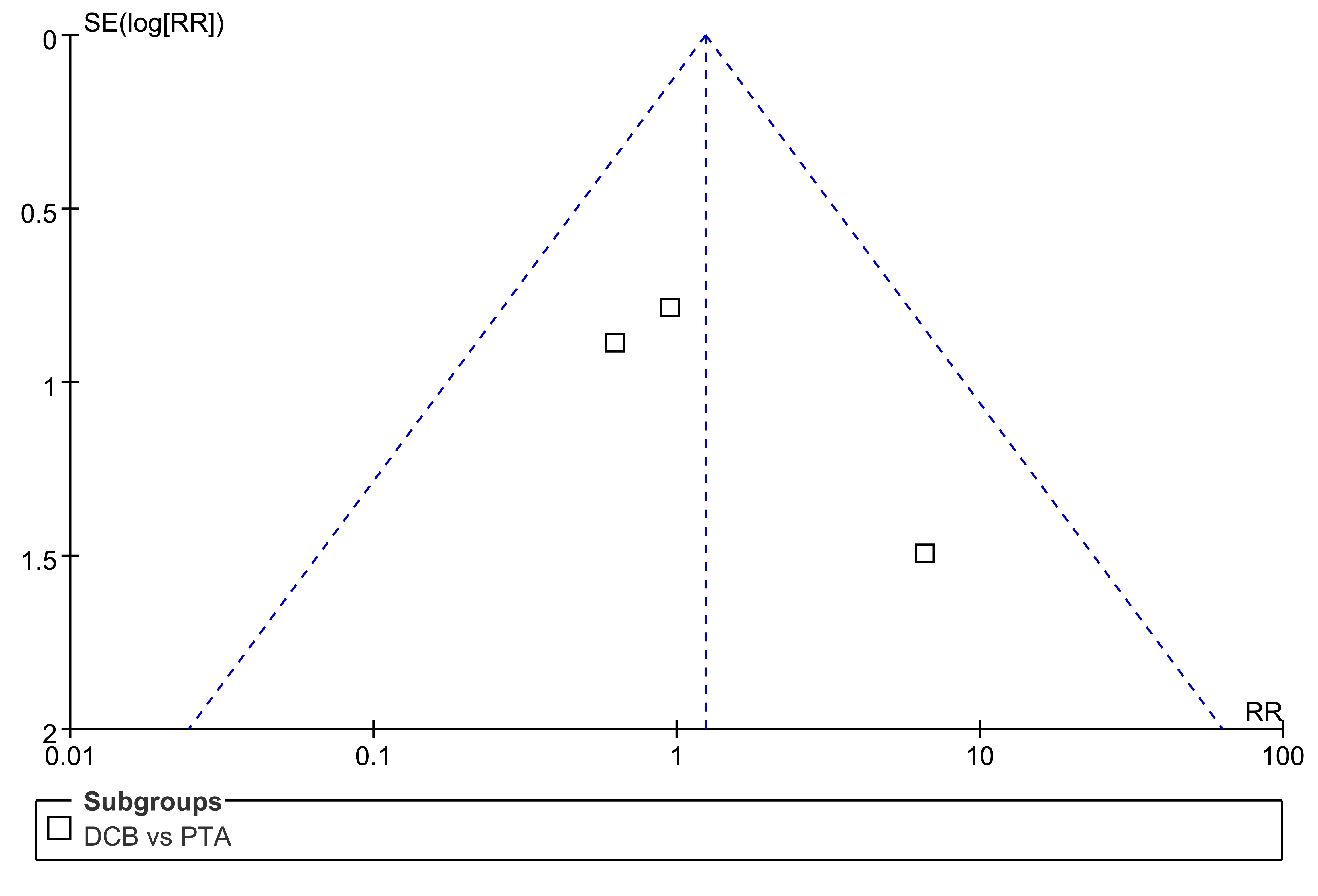


Figure S9 funnel plot of all-cause mortality


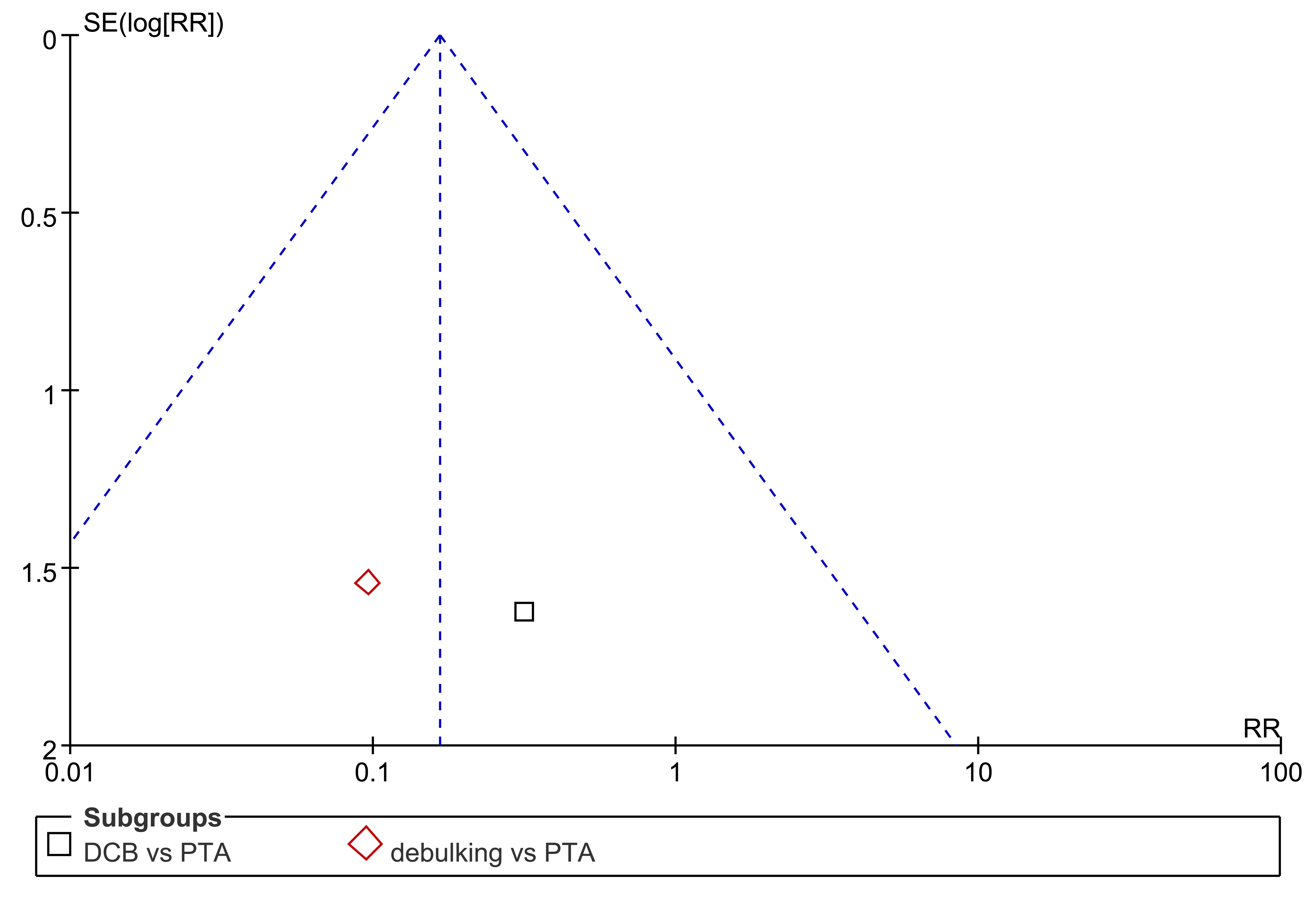


Figure S10 funnel plot of amputation

Table S1 8 full-text excluded articles

|  | Design | intervention | | Reasons for exclusion |
| --- | --- | --- | --- | --- |
|  |  | experiment | control |  |
| Dick, P. 2008 | single-site, prospective, randomized, controlled | PCBA | PTA | not DCB or debulking in the experiment group |
| Gandini, R. 2013 | single-site, prospective, randomized, controlled | LA+DCB | DCB | Combination therapy of LA and DCB in experiment group |
| Sixt, S. 2013 | retrospective analysis | DA+DCB | DA+PTA | Combination therapy and retrospective analysis |
| Armstrong, E. J. 2015 | retrospective analysis | LA | PTA | retrospective analysis |
| Grotti, S. 2016 | retrospective analysis | DCB | PTA | retrospective analysis |
| Murata, N. 2016 | retrospective analysis | DES | PTA | retrospective analysis and not DCB or debulking in the experiment group |
| Tomoi, Y. 2016 | retrospective analysis | DES | PTA | retrospective analysis and not DCB or debulking in the experiment group |
| Kokkinidis, D. G. 2018 | retrospective analysis | LA+DCB | LA+PTA | Combination therapy and retrospective analysis |

PCBA: peripheral cutting balloon angioplasty, PTA: percutaneous transluminal, LA: laser atherectomy, DCB: drug-coated balloon angioplasty angioplasty, DA: directional atherectomy, DES: drug-eluting stents.
